# Reconstruction of the urethra with a Surgisis^®^ onlay patch in urethral reconstructive surgery: two case reports

**DOI:** 10.1186/1752-1947-3-6542

**Published:** 2009-03-16

**Authors:** Thorsten H Ecke, Steffen Hallmann, Holger Gerullis, Jürgen Ruttloff

**Affiliations:** 1 Department of Urology, HELIOS Hospital Pieskower Strasse, Bad Saarow 15526 Germany; 2 Department of Urology, Lukas Hospital Preussenstrasse, Neuss 41464 Germany

## Abstract

**Introduction:**

We present two case reports of patients with recurrent stricture of the urethra. We used Surgisis^®^ for reconstruction.

**Case presentation:**

In these two case reports, we show the positive results of reconstructive surgery with Surgisis^®^ as an alternative surgical approach to common onlay patch surgery of the urethra performed on two Caucasian patients: a 48-year-old man and a 55-year-old man.

**Conclusion:**

Compared to buccal mucosa flap or foreskin graft surgeries for urethral reconstruction, reconstructive surgery with Surgisis^®^ is considered a relevant therapeutic alternative because of the shorter operation time and the preventable surgery of the buccal cavity or foreskin.

## Introduction

Urethral strictures are defined as restrictions of the urethral lumen irrespective of length and localization. Independent of its origin, diagnosis and treatment of a urethral stricture should be carried out as early as possible in order to avoid irreversible long-term damage [1-2].

Every process affecting the urethral urothelium and the covered tissue of the cavernous body may induce scarring, which can cause urethral stricture. Internal urethrotomy using the Sachse technique is a well established surgical approach for treatment of primary strictures.

Particularly for recurrent or long-segment strictures, open surgical approaches should be preferred because of the known lower relapse rate [2-4]. Widespread applications are in use for autologous transplants, such as urethroplasty with buccal mucosal free grafts [4,5]. Using biodegradable grafts is an excellent solution in this context. In animal studies, the experimental use of small intestinal submucosa (SIS) for reconstruction in the urinary tract has shown promising results [6,7]. The SIS is a collagen-based, nonimmunogenic material obtained from the submucosal layer of a pig's small bowel [5].

We report an alternative to urethroplasty with buccal mucosal free grafts, namely, open surgery urethral reconstruction using porcine small intestine submucosa (Surgisis®) as an onlay patch [8,9].

## Case presentation

Case 1: A 48-year-old Caucasian man presented to our institution in February 2004 with a short-segment bulbar urethral stricture. No previous history of trauma or sexually transmitted disease was reported at the time of presentation.

We initially performed urethrotomy using the Sachse technique without complications. Two years later, the patient complained again of decreasing urine stream and frequency.

**Figure 1.**
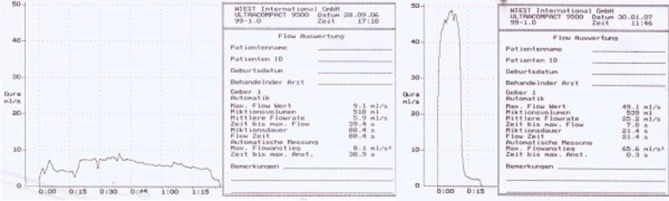
**(A)** and **(B)** Uroflowmetry before and after reconstruction (case 1).

As shown in Figure 1A, maximal flow was 9.1ml/sec (micturition volume 518ml, micturition time 88 seconds).

**Figures 2.**
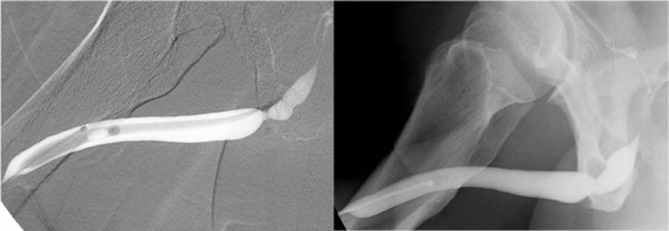
**(A)** and **(B)** Retrograde urethrography before and after reconstruction (case 1).

Retrograde urethrography revealed a recurrent urethral stricture, as shown in Figure 2A.

**Figures 3.**
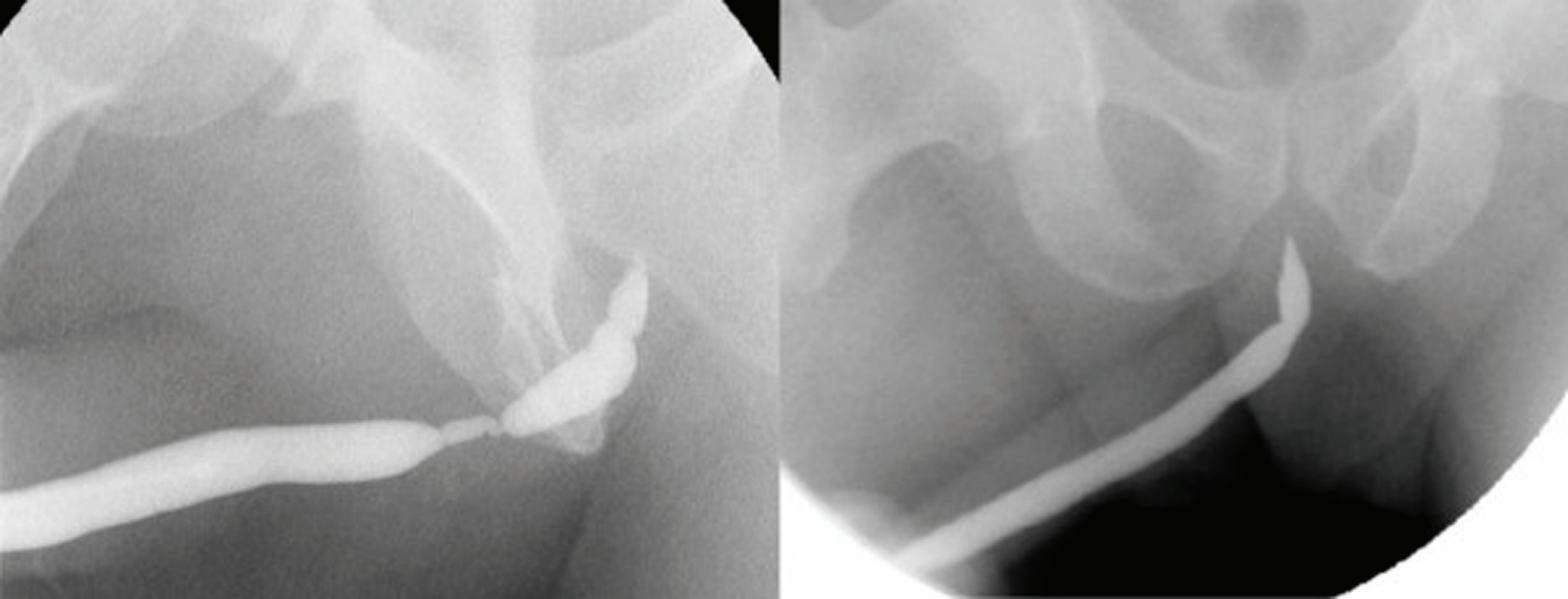
**(A)** and **(B)** Retrograde urethrography before and after reconstruction (case 2).

Case 2: Thirty-eight years before presentation, this 55-year-old Caucasian man had undergone an open urethral reconstruction after traumatic urethral damage. A recurrent stricture was treated with urethrotomy using a laser technique in 2006. A secondary recurrent urethral stricture developed during short-term follow up, as shown in Figure 3A.

Uroflowmetry revealed a maximal flow of 5.9 ml/second (micturition volume 489 ml, micturition time 120.6 seconds).

In both patients, open surgery urethral reconstruction using Surgisis® as onlay patch was performed in the dorsosacral position. The urethra was exposed and incised. We identified a 3 cm long stricture in our first patient, and a 4 cm long stricture in our second patient.

End-to-end anastomisis of the urethra was not possible in either case. After incision of the urethra 0.5 cm distally and proximally of the respective stricture, a Surgisis® patch was cut and inserted, in the same way as a buccal mucosal free graft would be inserted, using a 5 × 0 monofile thread with longitudinal splines, over a 16-Foley catheter.

The procedure included insertion of a suprapubic cystostomy. The operation time was 144 minutes for the first patient. Because of the complicated preparation, operation time was 162 minutes for the second patien.. No perioperative complications were seen in either case. The transurethral catheter was removed in both patients on day seven postoperatively, and both patients were treated with ciprofloxacin 2 × 500 mg postoperatively for eight days.

On day 24 after the operation, retrograde urethrography revealed good healing in both patients (Figures 2B and 3B). The percutanous cystostomy catheter was removed on day 25 in both patients.

Postoperative uroflowmetry performed on the first patient on day 25 revealed a maximal uroflow of 49.1ml/sec (micturition volume 539 ml, micturition time 21.4 seconds), as shown in Figure 1B. The same procedure performed on the second patient showed a maximal uroflow of 20.6ml/second (micturition volume 563 ml, micturition time 64 seconds), as shown in Figure 1B.

## Discussion

The choice of the appropriate material for reconstruction of the male urethra remains a focus of controversy [2,4,5,8,9]. Numerous surgical techniques have been previously described, and various types of autologous materials have been used in order to bridge urethral defects [1]. In some cases, the search for new applicable materials became mandatory because of the morbidity associated with classical approaches and the deficiency of available well-vascularized autologous tissues for urethral reconstruction [8,9]. The Surgisis® (by Cook Inc, Spencer, Indianapolis, USA) technique described here could be an interesting surgical alternative for recurrent strictures after previous open urethral surgery. This is one of the first reports in the medical literature of urethral surgery using Surgisis®.

Besides the use of Surgisis® in urethral reconstruction in rabbits with good results [5], synthetic grafts of silicone rubber, siliconized Dacron and Gore-Tex® have also been used for urethral reconstruction in animal experiments, but with poor results. Their use has been associated with a high incidence of infection, calcification and fistula formation [10].

Neither of our patients showed a significant lower flow after a median follow-up time of 22 months, and no further operation was necessary in either case. Neither patient showed complications of infection, allergic reaction, calcification or fistula. Furthermore, we found no probable atrophy of the newly applied tissue, and no recurrent urethral stricture was found.

Biodegradable grafts seem to be an ideal solution for the repair of the urethra as well as other segments of the urinary tract. SIS acts like a framework for the host-tissue cells to migrate and regenerate the organ, both in shape and in function [6,7]. We use Surgisis® to cover urethral defects. Calculating one minute of operation to cost around 15€ and the Surgisis® material used to cost around 280€, we believe that saving over 30 minutes of operation time will more than pay for the cost of use of the Surgisis® material [11].

Although the use of Surgisis® in urethral surgery is an interesting alternative to buccal mucosa flap or foreskin graft surgeries, further studies are needed to evaluate the value of this new technique. Comparison of implantation techniques, position of the graft, antibiotic prophylaxis, catheterization time and long-term outcome need to be documented. Until studies with Surgisis® have demonstrated superiority in efficacy and absence of side effects, buccal mucosa flap or foreskin graft surgery remain the first choices of treatment in patients with long bulbar or penile strictures [1-3,8].

## Conclusions

Application of the commercially provided implant system Surgisis® appears to be a reasonable alternative to buccal mucosa flap or foreskin graft surgery in urethral reconstructive surgery. An important advantage of Surgisis® is the prevention of the additional surgery needed in order to obtain a buccal mucosa or foreskin graft. Thus postoperative morbidity and overall surgery time decrease.

## Abbreviations

SIS: small intestinal submucosa
